# Endometrial scratching during hysteroscopy in women undergoing *in vitro* fertilization: a systematic review and meta-analysis

**DOI:** 10.3389/fsurg.2023.1225111

**Published:** 2023-09-19

**Authors:** Evangelos Papanikolaou, Nikolaos Peitsidis, Ioannis Tsakiridis, Georgios Michos, Antonios Skalias, Dimitrios Patoulias, Alexandros Poutoglidis, Apostolos Mamopoulos, Apostolos Athanasiadis, Grigorios Grimpizis, Robert Najdecki

**Affiliations:** ^1^ Assisting Nature IVF Centre and Genetics, Thessaloniki, Greece; ^2^ 3rd Department of Obstetrics and Gynaecology, General Hospital Hippocrateion, Aristotle University of Thessaloniki, Thessaloniki, Greece; ^3^ 2nd Academic Otorhinolaryngology, Head and Neck Surgery Department, Papageorgiou Hospital, Aristotle University of Thessaloniki, Thessaloniki, Greece; ^4^ Department of Obstetrics and Gynaecology, Interbalkan Hospital, Thessaloniki, Greece; ^5^ Department of Otorhinolaryngology, Head and Neck Surgery, “G. Papanikolaou” General Hospital, Thessaloniki, Greece; ^6^ 1st Department of Obstetrics and Gynaecology, Papageorgiou Hospital, Aristotle University of Thessaloniki, Thessaloniki, Greece

**Keywords:** hysteroscopy, endometrial scratching, IVF, repeated implantation failure, reproductive outcomes

## Abstract

**Objective:**

Endometrial scratching (ES) during hysteroscopy before embryotransfer (ET) remains doubtable on whether it benefits the reproductive outcomes. The optimal technique is not clear and repeated implantation failure as a challenging field in *in vitro* fertilization (IVF) seems to be the springboard for clinicians to test its effectiveness.

**Methods:**

Medline, PMC, ScienceDirect, Scopus, CENTRAL, Google Scholar were searched from their inception up to April 2023 for studies to evaluate the effectiveness of adding endometrial scratching during hysteroscopy before ET.

**Results:**

The initial search yielded 959 references, while 12 eligible studies were included in the analyses, involving 2,213 patients. We found that hysteroscopy and concurrent ES before ET resulted in a statistically significant improvement in clinical pregnancy rate (CPR) [RR = 1.50, (95% CI 1.30–1.74), *p <* 0.0001] and live birth rate (LBR) [RR = 1.67, (95% CI 1.30–2.15), *p *< 0.0001] with no statistically significant difference on miscarriage rate [RR = 0.80 (95% CI 0.52–1.22), *p* = 0.30]

**Conclusion:**

Our meta-analysis suggests that hysteroscopy with concurrent ES may be offered in IVF before ET as a potentially improving manipulation. Future randomized trials comparing different patient groups would also provide more precise data on that issue, to clarify specific criteria in the selection of patients.

**Systematic Review Registration:**

PROSPERO (CRD42023414117)

## 1. Introduction

Hysteroscopy has been rapidly spread in *in vitro* fertilization (IVF) as it seems to improve the chances of clinical pregnancy or live birth (1). Many reproductive medicine specialists recommend hysteroscopy as an accurate tool compared to the high false-positive and false-negative rates in detecting intrauterine abnormalities with hysterosalpingography (HSG) (2–4). Nevertheless, World Health Organization (WHO) recommends office hysteroscopy only in cases where an intrauterine abnormality is suspected by other clinical or complementary diagnostic exams (3).

Improving embryo quality and endometrial receptivity emerging research as the most important factors for successful implantation, which still remains a rate-limiting step in assisted reproductive technology (ART) (5). An intentional endometrial scratching caused by a pipelle biopsy or curettage is defined as endometrial scratching (ES) and potentially enhances embryo implantation through the improvement of endometrial receptivity (6). Endometrium and embryo synchronicity improvement, induction of endometrial decidualization and histamine release during endometrial scratching are several theories proposed to explain how ES may facilitate endometrial receptivity (7–9).

Combining hysteroscopy with ES seems to be a new trend in IVF as many authors have published trials with favorable results after this manipulation (3, 10–20). However, a reported significant variation exists in the patient population (unselected, one or more IVF failures), scratching technique (plastic biopsy catheter, Novak curette, hysteroscopic scissors, claw forceps, or the scope itself) and the timing of the scratching [early or late follicular phase before embryotransfer (ET)].

Thus, the aim of the present systematic review was to identify, critically appraise and summarize all the available relevant studies, in order to provide precise effect estimates on the impact of ES, during hysteroscopy, on pregnancy rates.

## 2. Methods

This systematic review and meta-analysis has been conducted according to the Preferred Reporting Items for Systematic Reviews and Meta-analyses (PRISMA) guidelines (21). No modifications were made to our protocol, which was prospectively registered in PROSPERO (CRD42023414117) and aimed to provide the most informed answer to our clinical question. Since the data used in this investigation were already published, no patient consent nor ethical approval was necessary.

### 2.1. Eligibility criteria

A search was conducted for studies with a minimum duration of 6 months and enrolling adult women <50 years old with intervention not in the same cycle of ovarian stimulation. The included studies could be randomized, prospective non-randomized, prospective, or retrospective. The intervention group included subfertile women who had undergone hysteroscopy with any type of ES compared to women with no intervention or hysteroscopy without ES before ET with fresh or frozen embryos.

No search restrictions were imposed as regards study design (parallel, crossover), study blinding (single-blind, double-blind, open-label), setting, and sample size. Reviews, case reports, published abstracts, congress abstracts and meta-analyses were excluded.

Study selection and data extraction were performed by two authors (N.P. and I.T.—both biostatisticians) independently; all articles including abstracts from the electronic searches were assessed and citations that met the initial predefined selection criteria were obtained. Study quality assessment and final inclusion/exclusion decisions were made after the examination of full manuscripts. After an independent assessment of the manuscripts, any disagreement between the two reviewers was resolved by consultation with a third reviewer (E.P.).

### 2.2. Information sources and search strategy

A systematic search was performed in major electronic databases, Medline, PMC, ScienceDirect, Scopus, the Cochrane Central Register of Controlled Trials (CENTRAL), and Google Scholar, for eligible studies from their inception up to April 2023. MeSH terms were used for both intervention and underlying disease, along with free-text words and the Boolean operators “OR” and “AND” were also used. Our search was restricted to human studies, although no filter was imposed regarding language or text availability.

The search terms for each database are provided at Supplementary Table S1.

### 2.3. Study selection and data collection process

All retrieved reports were imported into Mendeley, a reference manager software program for deduplication. Any remaining reports were then reviewed at a title and abstract level by two independent reviewers (N.P. and I.T.) and all potentially eligible studies were full text assessed. We also extracted data about study features (design, country, and time period of the study), population (number of patients and inclusion criteria), type of intervention (timing and instruments), *in vitro* fertilization embryotransfer (IVFET) cycles (ovarian stimulation protocols, drugs for endometrial preparation, embryos transferred, luteal-phase support) and study outcomes. Any disagreements between the two reviewers at any stage were resolved by discussion, consensus or arbitration by a third senior reviewer (E.P.). When insufficient information was reported in the articles, as well as when only a recorded study protocol was identified, we contacted authors (by e-mail) to ask for further data.

### 2.4. Data items

The main outcomes of this study were clinical pregnancy rate (CPR) and live birth rate (LBR). Additionally, data concerning miscarriage rate (MR) and beta-human chorionic gonadotrophin (bHCG) were extracted and synthesized.

LBR (per patient): “Ongoing pregnancy” defined as a pregnancy beyond 12 weeks of gestation. “Live birth” defined as the delivery of one or more living infant(s) after 24 weeks' gestation and surviving for at least 1 month.

CPR (per patient): Defined as the presence of a gestational sac on transvaginal ultrasound 6–8 weeks after ET or other definitive clinical signs.

MR (per clinical pregnancy): Defined as fetal loss before the 20th week of gestation.

### 2.5. Risk of bias assessment

Two authors (N.P. and I.T.) independently assessed the risk of bias within studies by using the risk of bias tool outlined in the Cochrane Handbook for Systematic Reviews of Interventions, which was integrated into the Review Manager 5.4.1 software (22). Seven domains related to the risk of bias were assessed: random sequence generation; allocation concealment; blinding of participants and personnel; blinding of outcome assessment; incomplete outcome data; selective data reporting; and other bias. Authors' judgments were reported as “low risk,” “high risk,” or “unclear risk” of bias. For the estimation of “selective data reporting,” we evaluated study protocols, when available. If not available, studies were judged as unclear risk of bias. Results were compared, and disagreements were discussed with a third reviewer (D.P.).

### 2.6. Effect measures and synthesis' methods

Study features and outcomes were assembled in a tabular form, and formal meta-analysis was performed using RevMan 5.4.1 software (22), with *P* < 0.05 set as the level of significance. A random-effects model (using the Mantel–Haenszel method) was used because of the difference in study designs and the method used for intervention (hysteroscopy and ES). The effect estimate was expressed as risk ratio (RR) with a 95% confidence interval (CI) and represented graphically by forest plots. Statistical heterogeneity was examined using the chi-squared test and I2. Further sensitivity analysis was performed to assess the heterogeneity and outcome differences between randomized and non-randomized studies. Additionally, subgroup analysis for women with repeated implantation failure (RIF) was performed.

### 2.7. Reporting bias assessment

To estimate and minimize reporting bias, we addressed the possibility of missing studies from the synthesis using funnel plots as appropriate.

### 2.8. Certainty assessment

The Grading of Recommendations Assessment, Development and Evaluation (GRADE) approach (23) was used to assess the credibility of our summary estimates. Two reviewers (D.P. and I.T.) graded the major safety and effectiveness outcomes for evidence of inconsistency, risk of bias, indirectness, imprecision and publication bias. Any disagreements between reviewers were resolved by discussion, consensus, or arbitration by a third senior reviewer (E.P.).

## 3. Results

### 3.1. Study selection

**Figure 1:**
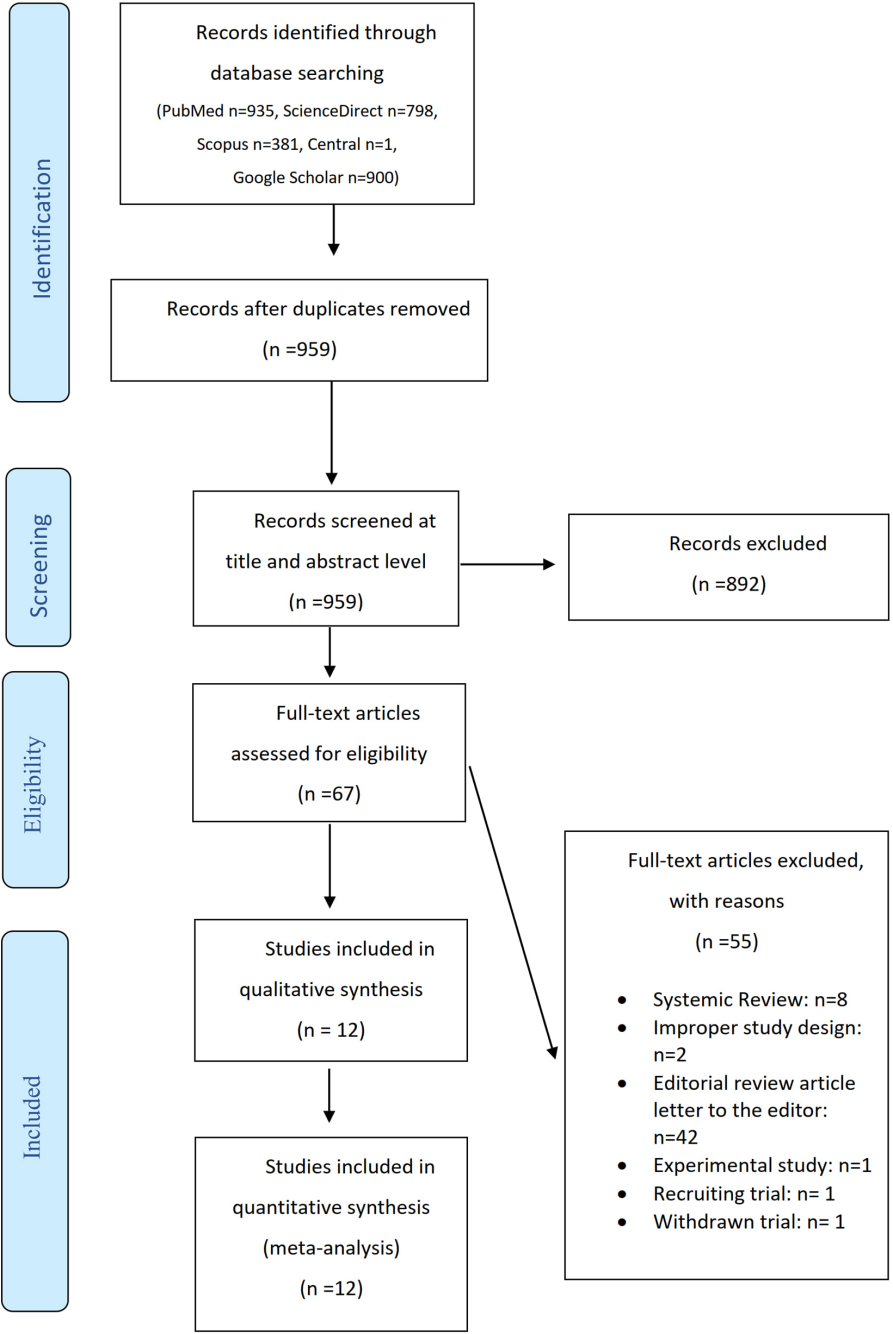
Flow diagram.

The initial database research identified 3,015 records; 959 records remained after removing duplicates. After screening their titles and abstracts, 892 records were excluded. The remaining 67 studies were reviewed in full text and 12 studies were finally considered eligible for inclusion in the qualitative and quantitative synthesis. The reasons for exclusion were improper study design, experimental study, recruiting trial, withdrawn trial, editorial review article/letter to the editor and systematic reviews. The flow diagram is shown in Figure 1 and the study characteristics are listed in Table 1. Of note, this subject was mostly researched in Egypt. Other countries include Greece, Turkey, Taiwan, Denmark, Hungary, and India. The instruments used were hysteroscopic scissors, claw forceps, biopsy catheter, sharp curette, monopolar diathermy with needle forceps, and the hysteroscope itself. The timing of ET varied among studies.

**Table 1:** Characteristics of included studies.

First author (year)	Setting	Study design	Intervention/control	Inclusion criteria	No. of participants (EI vs. no EI group)	IVF/ICSI	Instrument used	Timing of ES	Outcomes
Huang et al. (20)	Single center, Taiwan, March–October 2010	Proof of concept study	Site specific hysteroscopic biopsy vs. usual care	RIF (2 or more ET)	6–24	IVF	Claw forceps	D 4–7 of the ongoing hyperstimulation cycle	PR, IR, MR
Abdelrahman et al. (18)	Two centers, Egypt, July 2015–June 2016.	Prospective RCT	Guided hysteroscopy blunt scratching at lateral walls vs. usual care	One or more frozen embryo(s), normal uterine cavity (confirmed by vaginal ultrasonography)	45–45	IVF	Guided hysteroscopy using the right angle	Luteal phase preceding ET	ChP, IR, MR
Siristatidis et al. (16)	Two centers, Greece, March 2013–July 2016	Prospective nonrandomized	Three cuttings 1 cm lower on the front endometrial wall during hysteroscopy	RIF (2 IVF attempts, in which ≥2 embryos of high-grade quality were transferred in each cycle), 25–42 years, BMI 35 and 19, normo-ovulatory	51–52	IVF-ICSI	Hysteroscopic scissor	D 6–9 of the previous to ET cycle	LBR, β-hCG, CPR, MR,
Abou Senna et al. (14)	Single center Egypt, (2018)	Prospective observational study	Single, site-specific endometrial scratching guided by hysteroscopy vs. usual care	Previous failed at least 1 IVF outcome, normal uterine cavity, ≤37 years, normal HSGA + TVS	50–50	IVF-ICSI	Biopsy catheter	D 7–10 of the cycle prior to the embryo transfer	PR, IR, CPR
Gurgan et al. (13)	Private center, Turkey, February 2015, and October 2017	Prospective RCT	Cuttings with scissor into endometrium of fundus, posterior and anterior wall during hysteroscopy vs. usual care	RIF (least 4 good-quality embryos in a minimum of 3 fresh or frozen–thawed ET cycles), <40 years, FSH <15 IU/ml, BMI 18, 5–30, normal uterine cavity	124–115	IVF	Hysteroscopic scissor	D 10–12 of the late follicular phase in the preceding cycle	LBR, CPR, IR,
Berntsen et al. (12)	Two centers Denmark, 2013–2018	RCT with no blinding	Posterior wall of the uterus and with two biopsies in total during office hysteroscopy vs. usual care	Previous 1 failed IVF outcome, 18–40 years BMI <35, normal uterine cavity	92–92	IVF/ICSI	7 F forceps (GIMMI1 GmbH	Follicular phase of the cycle preceding stimulation	LBR, β-hCG, OP,
Acet et al. (10)	Single center, Turkey, 2014–2019	Retrospective cohort study	Hysteroscopy and endometrial scratching with gentle conventional curettage vs. usual care	RIF, (2 unsuccessful IVF/ET cycle performed, <40 years)	225–125	IVF	Sharp curette	Early follicular phase of the menstrual cycle prior to the subsequent IVF/ET cycle	LBR, PR, CPR
Kovacs et al. (15)	Single center, Hungary, August 2012–August 2013	Retrospective matched cohort	Diagnostic hysteroscope was gently pushed into the endometrium to disrupt it vs. usual care	RIF (≥2 IVF failures), ≤42 years, no intracavitary endometrial pathology	31–44	IVF	Diagnostic hysteroscope	Late follicular, early luteal phase of the menstrual cycle	IR, OPR, PR
Hafez et al. (11)	Two centers Egypt, June 2016–July 2018	Retro-prospective, control group-data based, intervention groups-prospective	As three cuttings of 0.5 cm on the front endometrial wall, 1 cm lower of the endometrial fundus level using a scissor, usual care	Undergoing the first ICSI cycle, 20–40 years, normal uterine cavity	75–75	ICSI	Hysteroscopic scissor	D 7–14 cycle before ET	PPT, CPR, MR, OPR, ChP
Shohayeb et al. (19)	Two canters Egypt, Saudi Arabia (2012)	Prospective randomized control trial	Hysteroscopy and endometrial scraping vs. hysteroscopy	RIF (two or more failed ICSI cycles despite the transfer of high-quality embryos), normal uterine cavity	100–100	ICSI	Novak Curette	D 4–7 follicular phase	LBR, IR, CPR, MR
Jayakrishnan et al. (3)	Single center India, June 2012–May 2015	Prospective cohort study	Hysteroscopy and endometrial scraping vs. hysteroscopy	The majority had one previous failed. IVF outcome	175–172	IVF/ICSI	Scope itself	Same cycle	CPR, IR
Seval et al. (17)	Single center Turkey, January 2011–June 2015	Retrospective cohort study	Scratching by monopolar electric energy with needle forceps during hysteroscopy vs. hysteroscopy	RIF (2 consecutive cycles of IVF, ICSI, or frozen embryo replacement cycles), without endometrial or uterine pathology, 20–40 years	171–174	IVF-ICSI	Monopolar electric energy with needle forceps during hysteroscopy	7–14 days prior to the subsequent ART cycle	IR, CPR, OP, MR

ART, assisting reproductive technics; ChP, chemical pregnancy; CP, clinical pregnancy rate; D, days; ICSI, intracytoplasmic sperm injection; IR, implantation Rate; IVF, in vitro fertilization; MR, miscarriage rate; OPR, ongoing pregnancy; RIF, repeated implantation failure; PPT, positive pregnancy test.

### 3.2. Risk of bias

**Figure 2:**
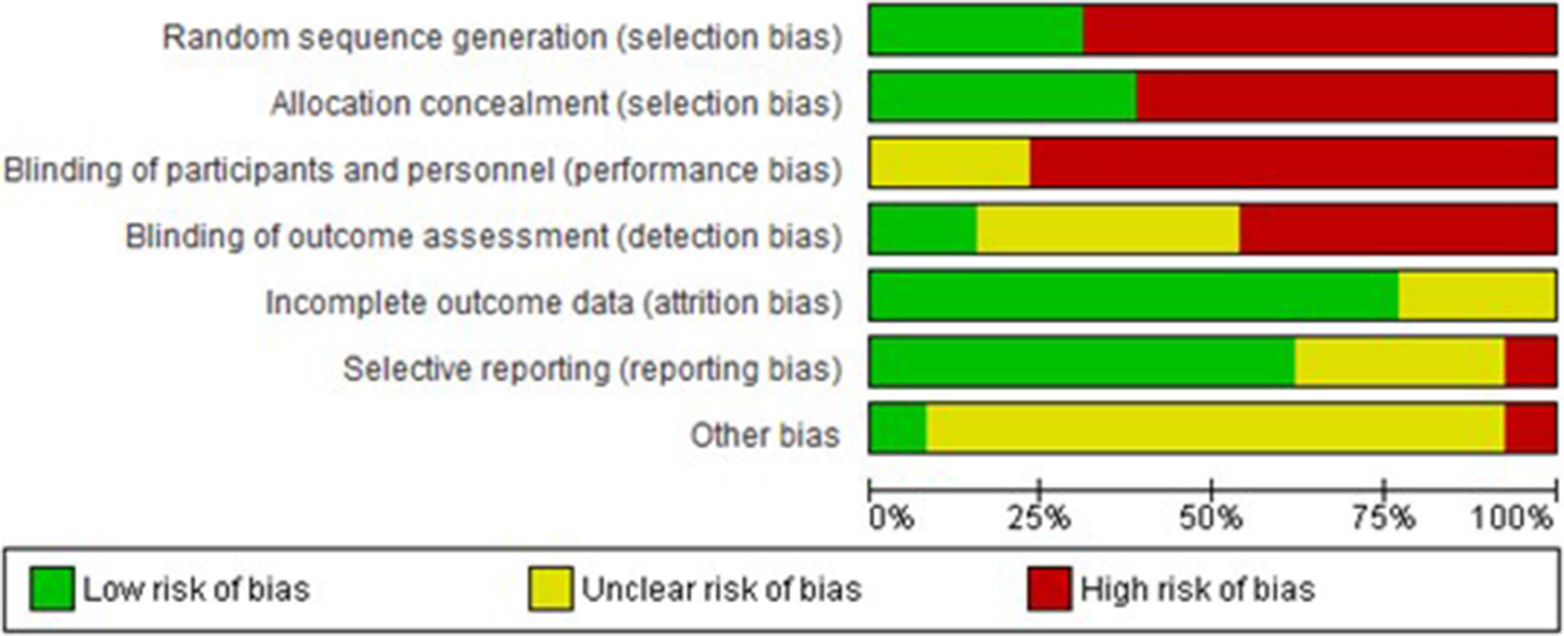
Risk of bias for each domain.

**Figure 3:**
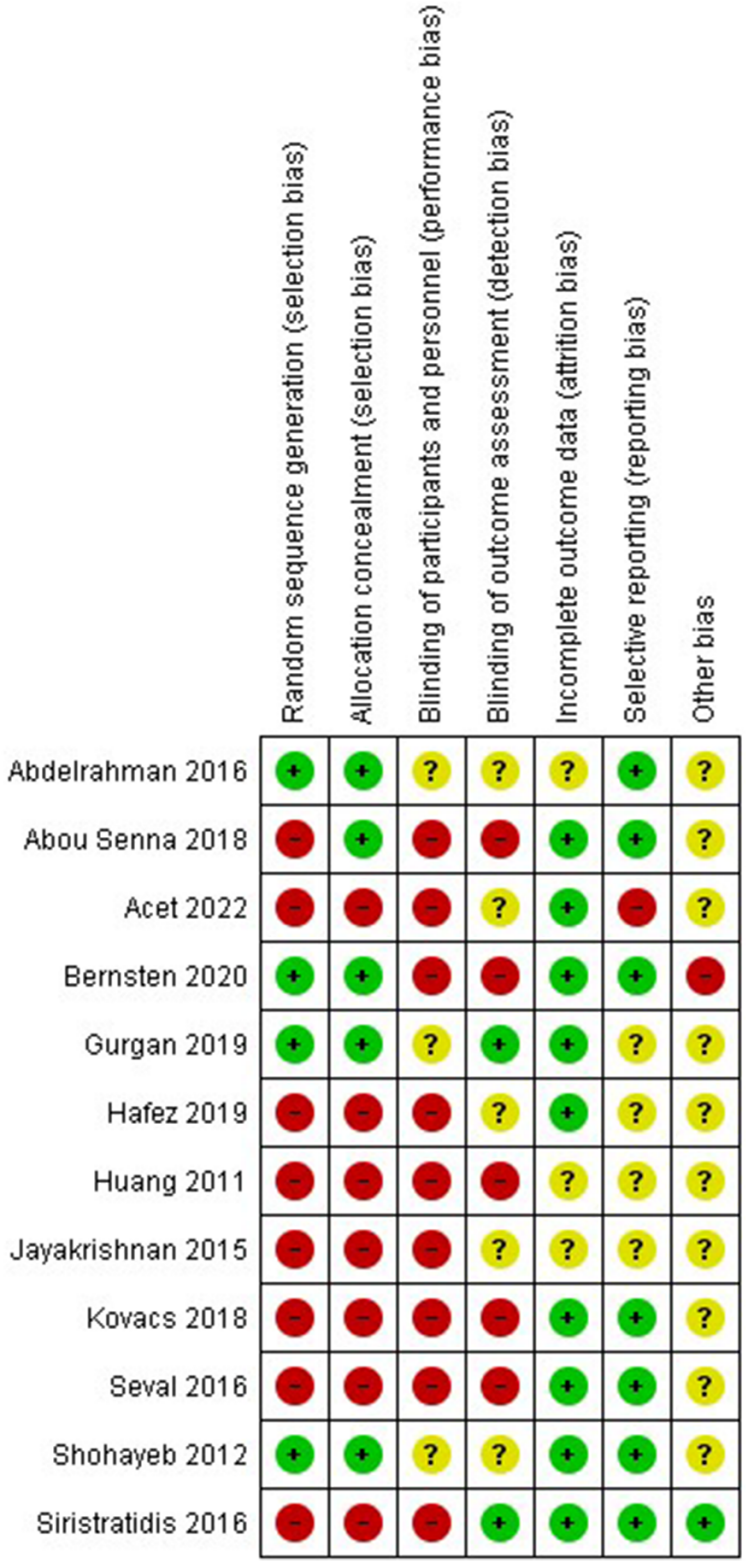
Risk of bias for each study.

The risk of bias is represented with a “traffic light” plot for each domain and each individual study is provided in Figures 2, 3, respectively. Additionally, due to the large number of meta-analyses undertaken and the fact that different studies were included in each analysis, the risk of bias for each study is also provided in the corresponding forest plot figure.

The search in **sources outside of published studies** did not retrieve additional studies.

The risk of unpublished results in published studies (**known unknowns**) appears to be small. In all studies identified, the authors provided data on the effectiveness of the method. In two studies there were no data on complications, while in one complications of sialendoscopy were not reported separately for sialolithiasis. However, the careful examination of the studies, as well as the fact that the safety of sialendoscopy was not their main outcome, do not raise suspicions about the possibility of risk of bias.

### 3.3. Certainty of evidence

The guidelines of the GRADE (Grades of Recommendation, Assessment, Development and Evaluation) framework were used to assess the certainty of evidence. The results are listed in the Summary of Findings table (Table 2).

**Table 2:** Summary of findings.

Outcomes	Results (RR)	95% confidence interval	Number of cases (studies)	Certainty of evidence
CPR	1.50	1.30–1.74	1,701 (9)	⊕⊕⊕O moderate
LBR	1.67	1.30–2.15	1,076 (5)	⊕⊕⊕O moderate
MR	Not statistically significant		483 (8)	⊕⊕OO low
bHCG	1.62	1.17–2.24	1,638 (9)	⊕⊕OO low

Endometrial scratching during hysteroscopy in women undergoing *in vitro* fertilization treatment

Population: women undergoing *in vitro* fertilization treatment

Intervention: endometrial scratching during hysteroscopy

### 3.4. Results of syntheses

**Figure 4:**
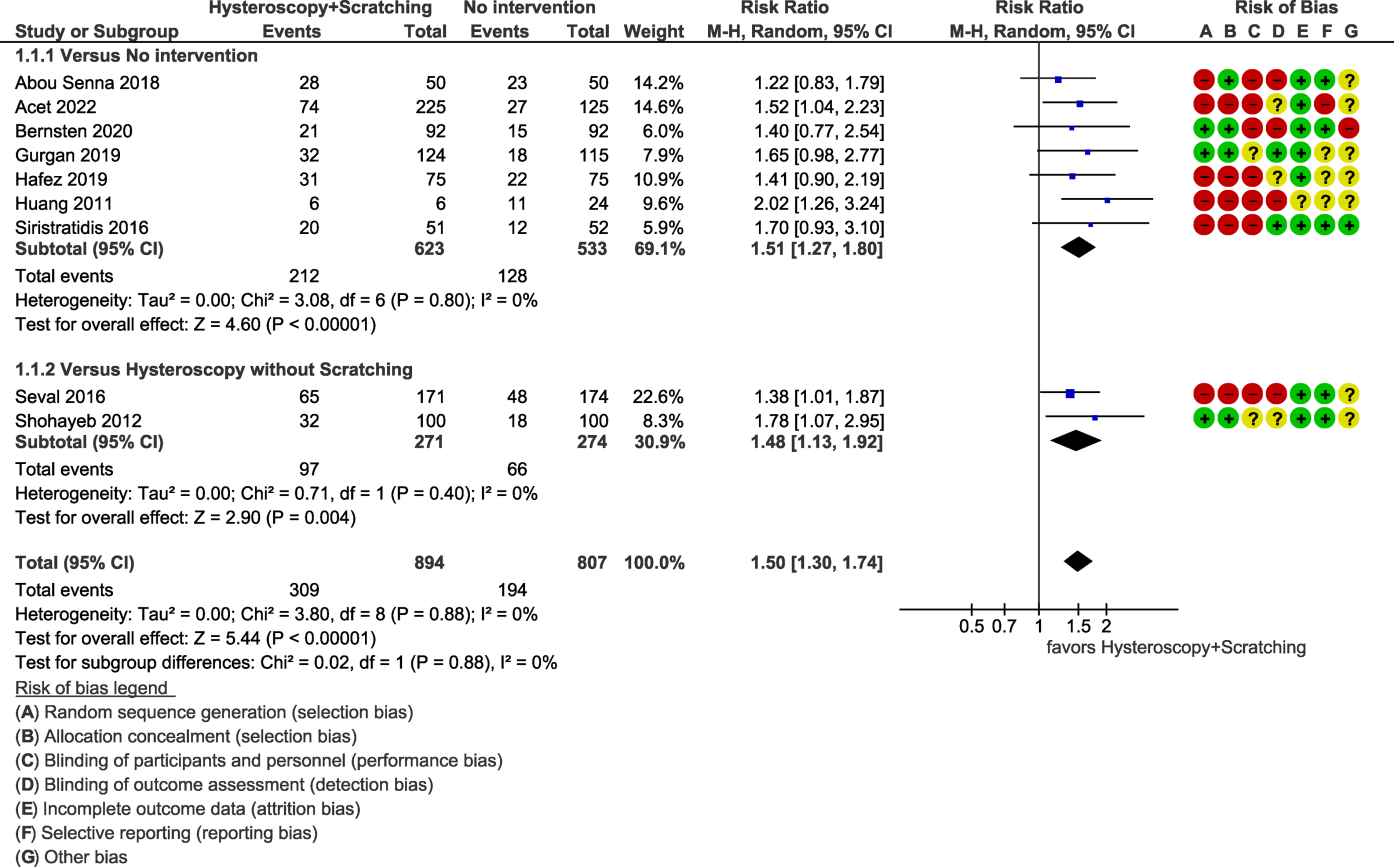
Meta-analysis of clinical pregnancy rate.

**Figure 5:**
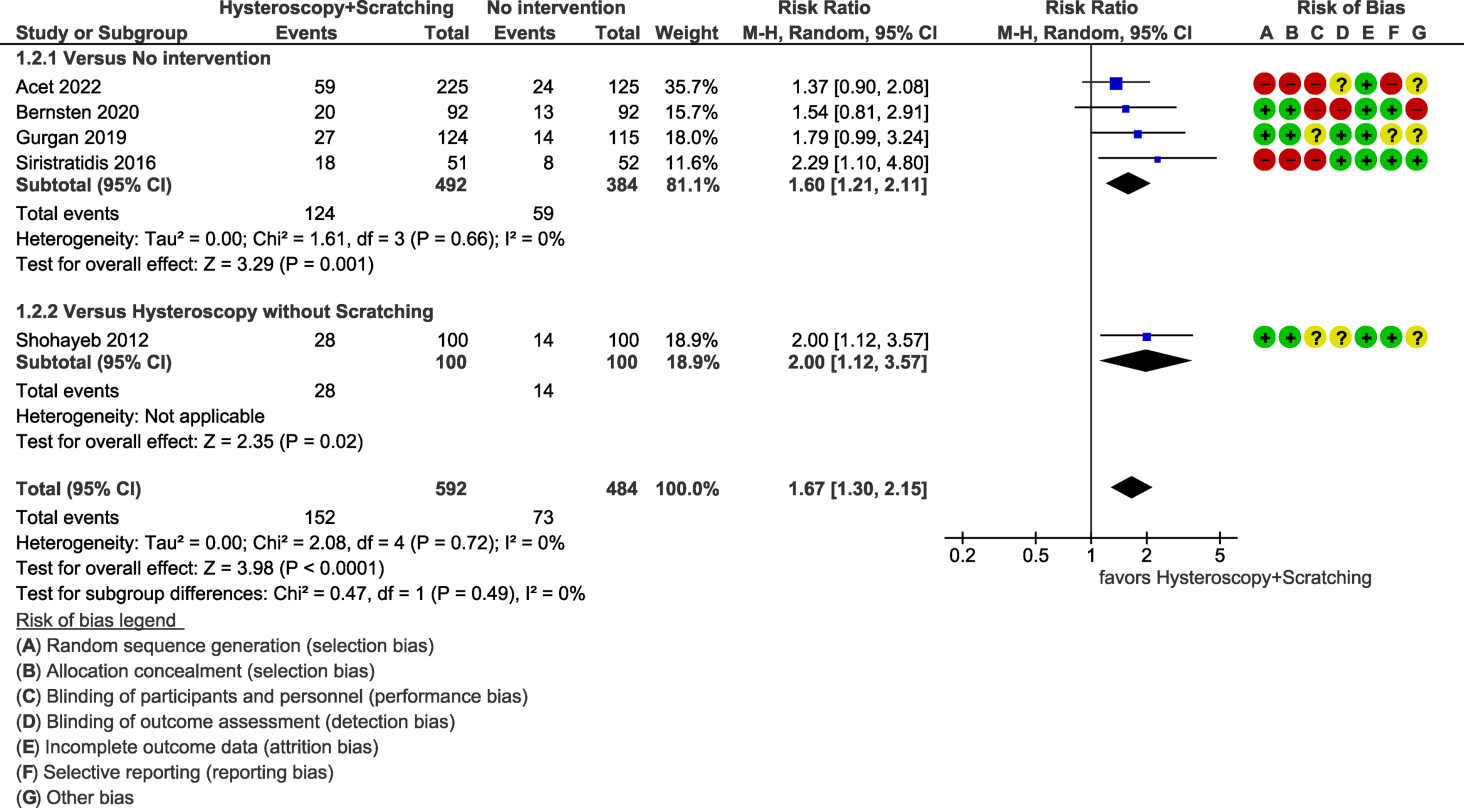
Meta-analysis of live birth rate.

**Figure 6:**
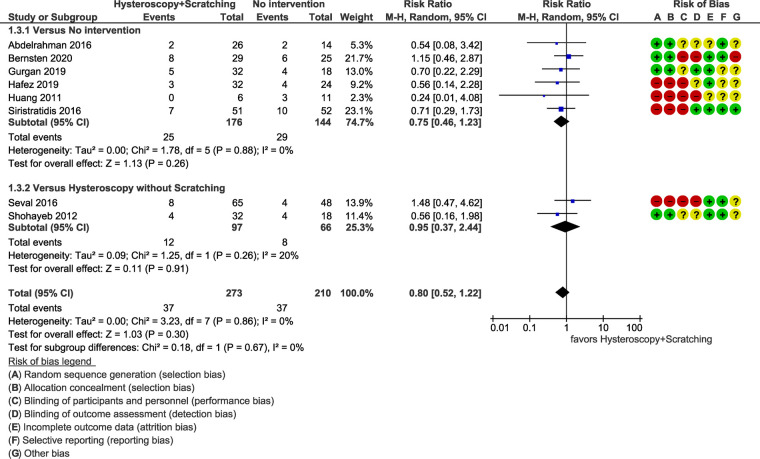
Meta-analysis of miscarriage rate.

**Figure 7:**
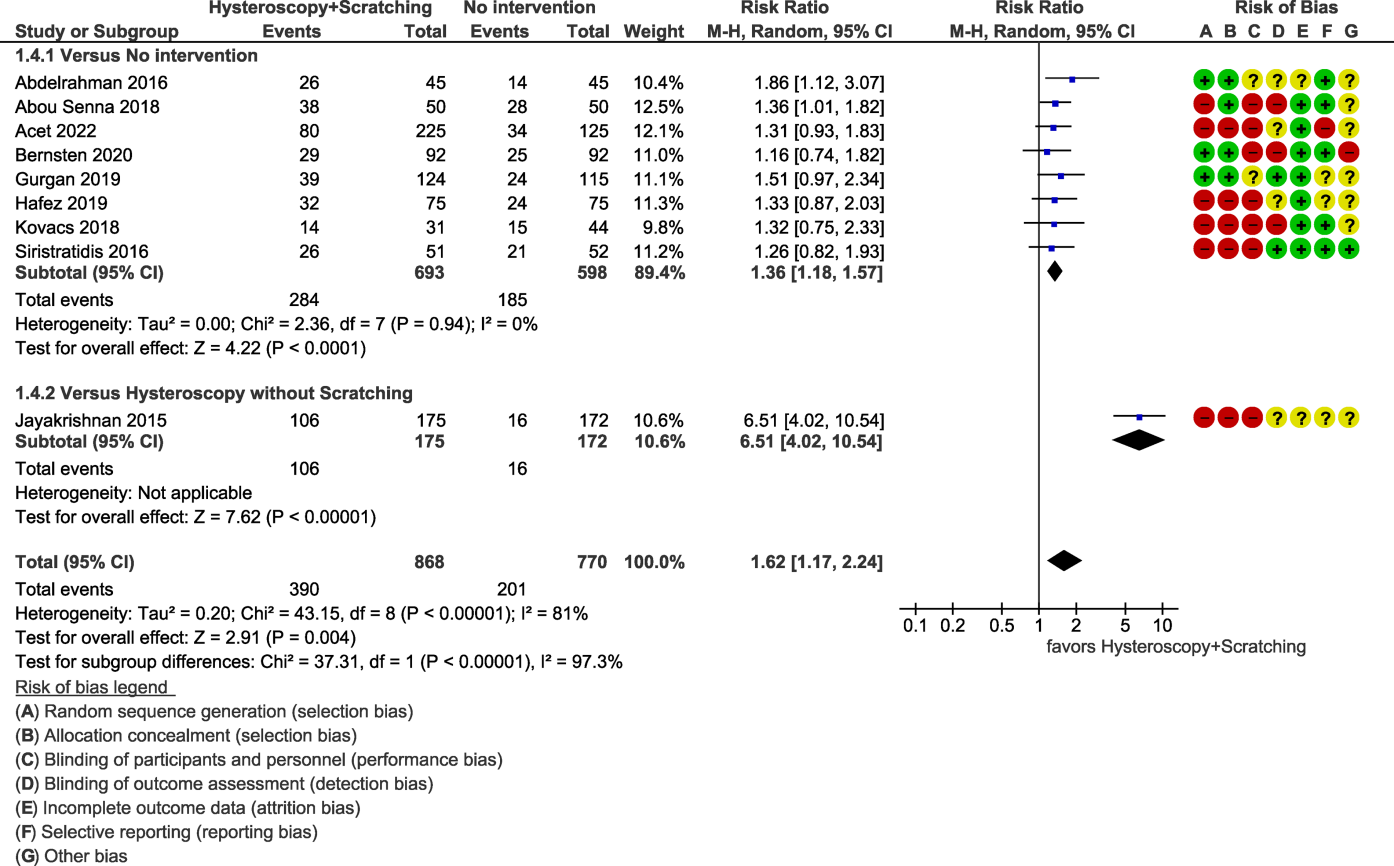
Meta-analysis of pregnancy indicated by bHCG.

The results of the studies comparing hysteroscopy combined with ES to no intervention or hysteroscopy without scratching, are listed below as forest plots (Figures 4–7). The measured outcomes were CPR, LBR, MR, and bHCG levels. Additionally, the forest plots for the sensitivity analysis including only randomized controlled trials (RCTs) are provided as Supplementary Figures S1–S4). The forest plots for the subgroup analysis for RIF patients are provided as Supplementary Figures S5–S8), as well.

#### 3.4.1. Clinical pregnancy rate

Nine studies, including a total of 1,701 patients, compared the outcome of hysteroscopy with scratching to no intervention or hysteroscopy alone. It was found that hysteroscopy combined with ES has significantly superior results [RR = 1.50 (95% CI 1.30–1.74), *p *< 0.0001] (Figure 4).

The heterogeneity of the studies was not statistically significant (*p *= 0.88 > 0.10 for Chi^2^ test, *I*^2^*^ ^*= 0%). Further sensitivity analysis including only RCTs (three studies, 623 patients) resulted in a RR of 1.62 (95% CI 1.19–2.21, *p = *0.001), which is in accordance with the result of all studies (Supplementary Figure S1).

Moreover, subgroup analysis for RIF patients (six studies, 1,267 patients) resulted in a similar outcome [RR = 1.59 (95% CI 1.33–1.90), *p *< 0.0001, *I*^2^*^ ^*= 0%] (Supplementary Figure S5).

#### 3.4.2. Live birth rate

Quantitative synthesis of data regarding LBR included five studies with a total of 1,076 patients. A statistically significant superiority of hysteroscopy combined ES for LBR was demonstrated [RR = 1.67 (95% CI 1.30–2.15), *p *< 0.0001] (Figure 5).

The heterogeneity of the studies was not statistically significant (*p *= 0.72 for Chi^2^ test, *I*^2^*^ ^*= 0%). Sensitivity analysis including only RCTs (three studies, 623 patients) resulted in a RR of 1.78 (95% CI 1.26–2.52, *p = *0.001) (Supplementary Figure S2).

RIF patients had a similar outcome [RR = 1.69 (95% CI 1.29–2.23), *p = *0.0002, *I*^2^*^ ^*= 0%] (four studies, 892 patients) (Supplementary Figure S6).

#### 3.4.3. Miscarriage

Miscarriage rates were reported in eight studies (483 patients). No statically significant difference was found among patients who underwent ES and those who did not [RR = 0.80 (95% CI 0.52–1.22), *p *= 0.30] (Figure 6).

The heterogeneity of the studies was not statistically significant (*p *= 0.86 for Chi^2^ test, *I*^2^*^ ^*= 0%). Sensitivity analysis including only RCTs (four studies, 194 patients) and subgroup analysis for RIF patients (five studies, 333 patients) did not reveal a statistically significant difference, as well (*p *= 0.46, *I*^2^*^ ^*= 0%), (*p *= 0.12, *I*^2^*^ ^*= 0%) respectively (Supplementary Figures S3, S7 respectively).

#### 3.4.4. Positive pregnancy test

For pregnancy detected with the use of bHCG, nine studies, including a total of 1,638 patients, were synthesized. The results indicated a significant superiority of hysteroscopy combined with ES [RR = 1.62 (95% CI 1.17–2.24), *p *= 0.004] (Figure 7).

However, the heterogeneity of the studies was statistically significant (*p *< 0.00001 for Chi^2^ test, *I^2 ^*= 81%). The reason is obvious since there is no statistically significant heterogeneity among the first subgroup (*p = *0.94 for Chi^2^ test, *I^2 ^*= 0%) and the second subgroup consists of a single study. Thus, we can assume that the heterogeneity stems from the differences between these two subgroups and no further investigation is required. However, caution is warranted in the interpretation of the results. We suggest using the results of each subgroup separately, rather than the pooled effect, keeping in mind that the result regarding the comparison to hysteroscopy alone is based on a single study. Furthermore, sensitivity analysis including only RCTs (three studies, 513 patients) resulted in a RR of 1.46 (95% CI 1.12–1.90), which is similar to the initial result (Supplementary Figure S4).

The subgroup analysis for RIF patients also demonstrated similar results [RR = 1.34 (95% CI 1.09–1.66), *p = *0.006, *I^2 ^*= 0%] (Supplementary Figure S8).

### 3.5. Risk of reporting bias in syntheses

**Figure 8:**
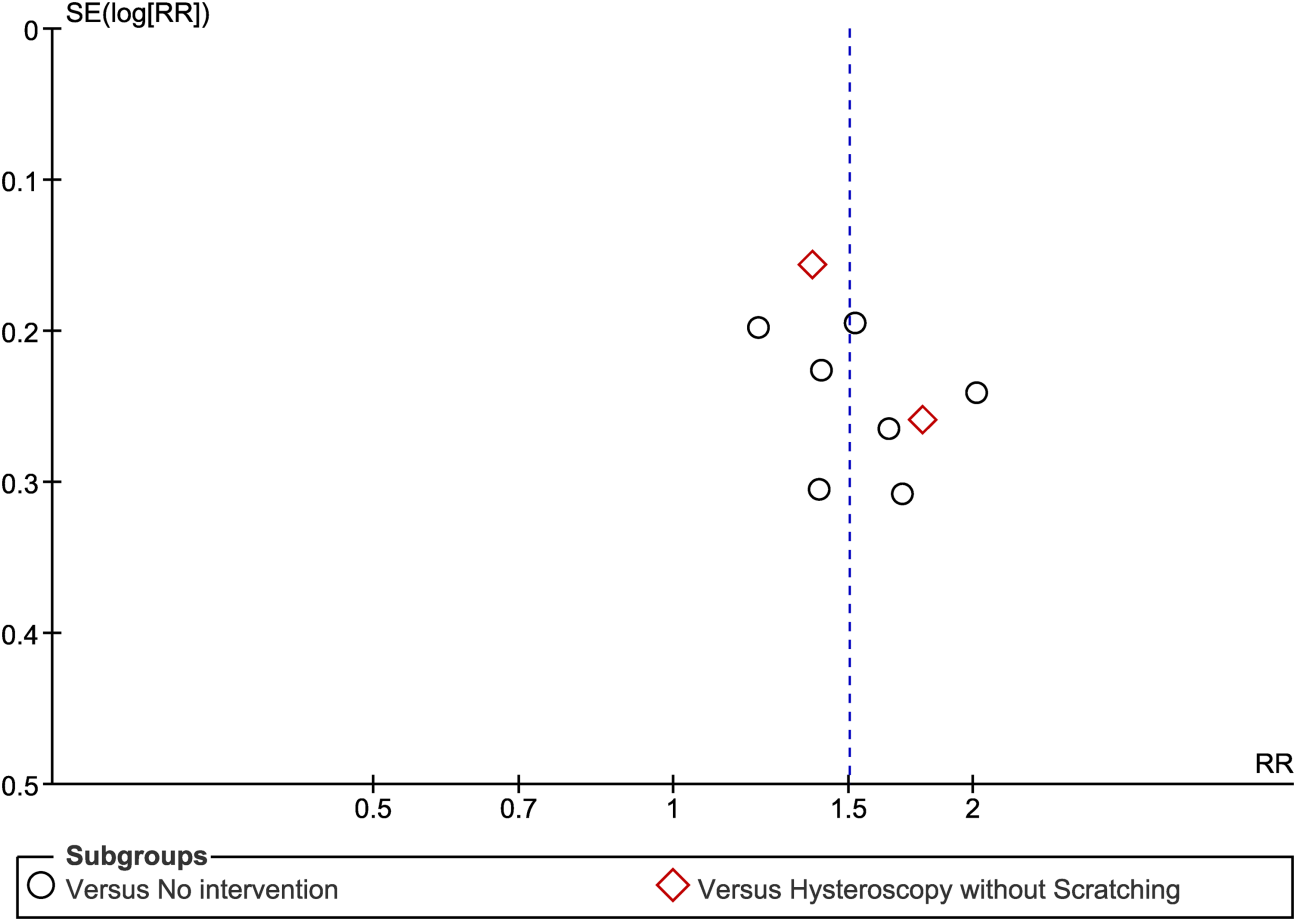
Risk of reporting bias funnel plot regarding the meta-analysis of clinical pregnancy rate.

We attempted to estimate the risk of reporting bias due to studies that have been undertaken but not reported. As a rule of thumb, tests for funnel plot asymmetry should be used only when there are at least 10 studies included in the meta-analysis. We created a funnel plot regarding the meta-analysis of clinical pregnancy rate, as it was the main outcome that included nine studies, which can be considered marginally acceptable (Figure 8). Visual inspection of the funnel plot gives a fairly symmetrical appearance, which can be interpreted as a low risk of reporting bias. However, especially in cases of few included studies, caution is warranted in the visual interpretation of funnel plots, while it always remains possible that the results are due to chance.

## 4. Discussion

According to the findings of the present meta-analysis, hysteroscopy with concurrent ES offered in IVF before ET is associated with higher clinical pregnancy and live birth rates, compared to no intervention or hysteroscopy alone.

The idea of endometrial scratching as a means of increasing pregnancy rates dates as far back as 1,907 when it was used in guinea pigs (17). In 2000 the hypothesis that endometrial scratching could increase pregnancy outcomes was incidentally formed (10, 24). This hypothesis was further explored, and the first study was reported in 2003, supporting the favorable outcomes of endometrial scratching especially using pipelle catheter (25). Since then, several more studies have been conducted using different ways of endometrial scratching (plastic biopsy catheter, Novak curette, pipelle, hysteroscopic scissors, claw forceps, the scope itself and different participant populations resulting in often contradictory outcomes (12, 26). Our systematic review and meta-analysis aim to provide a more solid answer to the specific question of the benefit of endometrial scratching during hysteroscopy in IVF.

Several mechanisms explaining the effect of scratching have been proposed. Decidualization of the endometrium or the secretion of cytokines and growth factors, as a result of the wound-healing process, have been described so far (27). The modulation in the expression of genes that may increase uterine receptivity is another plausible theory (28).

Additionally, the fact that ES during hysteroscopy can be performed as an easy additional procedure, renders it a cost-effective and appealing method for augmenting the chances of a successful IVF. Notably, despite common worries about the possibility of adverse effects of interventional techniques, the current literature does not raise worries and our results showed no statistically significant impact on miscarriage rates (3, 29).

The major limitation of this study is the poor quality of included studies, as indicated by the considerable risk of bias in many of them. Additionally, the studies included were limited in number, however, we should note that the number of patients included was adequate. Moreover, the timing of the intervention, as well as the instruments used, were different among the studies and this could potentially have an impact on the results.

The use of endometrial scratching during hysteroscopy before IVF seems to be an effective and safe method. It resulted in a statistically significant increase in CPR as well as LBR, nevertheless not reaching statistical significance in miscarriage rates. Additionally, its use has limited cost and does not require specific skills or instruments. Thus, the use of endometrial scratching in cases where hysteroscopy has been recommended before IVF may be considered as a routine procedure. Future randomized trials comparing different patient groups would provide more accurate data on this topic of interest.

## Data Availability

The original contributions presented in the study are included in the article/**Supplementary Material**, further inquiries can be directed to the corresponding author.

## References

[1] Kamath MS Bosteels J D'Hooghe TM Seshadri S Weyers S Mol BWJ Screening hysteroscopy in subfertile women and women undergoing assisted reproduction. Cochrane Database Syst Rev. (2019) 4(4):CD012856. 10.1002/14651858.CD012856.pub2 30991443 PMC6472583

[2] Campo R Meier R Dhont N Mestdagh G Ombelet W. Implementation of hysteroscopy in an infertility clinic: the one-stop uterine diagnosis and treatment. Facts Views Vis Obgyn. (2014) 6(4):235–9. 25593699 PMC4286863

[3] Jayakrishnan K Maya N Nambiar D. Impact on IVF outcome following Pre-IVF hysteroscopy and endometrial scratching. Obsgyne Rev J Obstet Gyneco. (2015) 1(1):21–6. 10.17511/joog.2015.i01.05

[4] Ahmadi F Rashidy Z Haghighi H Akhoond M Niknejadi M Hemat M Uterine cavity assessment in infertile women: sensitivity and specificity of three-dimensional hysterosonography versus hysteroscopy. Iran J Reprod Med. (2013) 11(12):977–82. 24639723 PMC3941409

[5] Nahshon C Sagi-Dain L Dirnfeld M. The impact of endometrial injury on reproductive outcomes: results of an updated meta-analysis. Reprod Med Biol. (2020) 19(4):334–49. 10.1002/rmb2.12348 33071635 PMC7542009

[6] Shoham G Alexandroni H Leong M Shulman A Weissman A. Use of endometrial scratching in IVF/IUI—a worldwide opinion and clinical practice survey. Clin Exp Obstet Gynecol. (2022) 49(5):108. 10.31083/j.ceog4905108

[7] Gnainsky Y Granot I Aldo PB Barash A Or Y Schechtman E Local injury of the endometrium induces an inflammatory response that promotes successful implantation. Fertil Steril. (2010) 94(6):2030–6. 10.1016/j.fertnstert.2010.02.022 20338560 PMC3025806

[8] Li R Hao G. Local injury to the endometrium: its effect on implantation. Curr Opin Obstet Gynecol. (2009) 21(3):236–9. 10.1097/GCO.0b013e32832a0654 19352180

[9] Shelesnyak MC. Inhibition of decidual cell formation in the pseudopregnant rat by histamine antagonists. Am J Physiol. (1952) 170(3):522–7. 10.1152/ajplegacy.1952.170.3.522 12985926

[10] Acet F Sahin G Goker ENT Tavmergen E. The effect of hysteroscopy and conventional curretage versus no hysteroscopy on live birth rates in recurrent in vitro fertilisation failure: a retrospective cohort study from a single referral centre experience. J Obstet Gynaecol. (2022) 42(6):2134–8. 10.1080/01443615.2022.2033963 35170394

[11] Hafez E Mahdy E AIbrahem M Ahmed M. Evaluation of endometrial scratching by hysteroscope or pipelle curette on the outcome of assisted reproduction. ZUMJ. (2020) 26(2):297–306.

[12] Berntsen S Hare KJ Lossl K Bogstad J Palmo J Praetorius L Endometrial scratch injury with office hysteroscopy before IVF/ICSI: a randomised controlled trial. Eur J Obstet Gynecol Reprod Biol. (2020) 252:112–7. 10.1016/j.ejogrb.2020.06.034 32593936

[13] Gurgan T Kalem Z Kalem MN Ruso H Benkhalifa M Makrigiannakis A. Systematic and standardized hysteroscopic endometrial injury for treatment of recurrent implantation failure. Reprod Biomed Online. (2019) 39(3):477–83. 10.1016/j.rbmo.2019.02.014 31405721

[14] Senna HFAA Gebreel MMI Ibraheem AHAE-H. Effect of adding endometrial scratching to hysteroscopy on pregnancy rates in women with recurrent implantation failure in IVF/ICSI cycles. Life Sci J. (2018) 15(12):34–44.

[15] Kovacs P Glenn T O’Leary K Lindheim S. Does endometrial injury increase the chance of pregnancy during IVF among patients with recurrent implantation failures? Gynecol Reprod Health. (2018) 2(1):1–6.

[16] Siristatidis C Kreatsa M Koutlaki N Galazios G Pergialiotis V Papantoniou N. Endometrial injury for RIF patients undergoing IVF/ICSI: a prospective nonrandomized controlled trial. Gynecol Endocrinol. (2017) 33(4):297–300. 10.1080/09513590.2016.1255325 27910711

[17] Seval MM Sukur YE Ozmen B Kan O Sonmezer M Berker B Does adding endometrial scratching to diagnostic hysteroscopy improve pregnancy rates in women with recurrent in-vitro fertilization failure? Gynecol Endocrinol. (2016) 32(12):957–60. 10.1080/09513590.2016.1190818 27258405

[18] Abdelrahman AH. Does endometrial injury guided hysteroscopy improve implantation rate of frozen-thawed embryo transfer? JMSCR. (2016) 04(12):15031–5. 10.18535/jmscr/v4i12.118

[19] Shohayeb A El-Khayat W. Does a single endometrial biopsy regimen (S-EBR) improve ICSI outcome in patients with repeated implantation failure? A randomised controlled trial. Eur J Obstet Gynecol Reprod Biol. (2012) 164(2):176–9. 10.1016/j.ejogrb.2012.06.029 22835632

[20] Huang SY Wang CJ Soong YK Wang HS Wang ML Lin CY Site-specific endometrial injury improves implantation and pregnancy in patients with repeated implantation failures. Reprod Biol Endocrinol. (2011) 9:140. 10.1186/1477-7827-9-140 22014336 PMC3210086

[21] Page MJ McKenzie JE Bossuyt PM Boutron I Hoffmann TC Mulrow CD The PRISMA 2020 statement: an updated guideline for reporting systematic reviews. Br Med J. (2021) 372:n71. 10.1136/bmj.n71 33782057 PMC8005924

[22] Cochrane. Review manager (RevMan). 5.4 ed. London: The Cochrane Collaboration (2020).

[23] Schünemann H Brożek J Guyatt G Oxman A. GRADE handbook for grading quality of evidence and strength of recommendations: the GRADE working group (2013). Available at: guidelinedevelopment.org/handbook

[24] Granot I Dekel N Bechor E Segal I Fieldust S Barash A. Temporal analysis of connexin43 protein and gene expression throughout the menstrual cycle in human endometrium. Fertil Steril. (2000) 73(2):381–6. 10.1016/S0015-0282(99)00531-2 10685547

[25] Barash A Dekel N Fieldust S Segal I Schechtman E Granot I. Local injury to the endometrium doubles the incidence of successful pregnancies in patients undergoing in vitro fertilization. Fertil Steril. (2003) 79(6):1317–22. 10.1016/S0015-0282(03)00345-5 12798877

[26] Metwally M Chatters R White D Hall J Walters S. Endometrial scratch in women undergoing first-time IVF treatment: a systematic review and meta-analysis of randomized controlled trials. Reprod Biomed Online. (2022) 44(4):617–29. 10.1016/j.rbmo.2021.11.021 35272939 PMC9089309

[27] Shokeir T Ebrahim M El-Mogy H. Hysteroscopic-guided local endometrial injury does not improve natural cycle pregnancy rate in women with unexplained infertility: randomized controlled trial. J Obstet Gynaecol Res. (2016) 42(11):1553–7. 10.1111/jog.13077 27363928

[28] Kalma Y Granot I Gnainsky Y Or Y Czernobilsky B Dekel N Endometrial biopsy-induced gene modulation: first evidence for the expression of bladder-transmembranal uroplakin ib in human endometrium. Fertil Steril (2009) 91(4):1042–9, 1049.e1-9. 10.1016/j.fertnstert.2008.01.043 18355812

[29] Bui BN Torrance HL Janssen C Cohlen B de Bruin JP den Hartog JE Does endometrial scratching increase the rate of spontaneous conception in couples with unexplained infertility and a good prognosis (hunault >30%)? Study protocol of the SCRaTCH-OFO trial: a randomized controlled trial. BMC Pregnancy Childbirth. (2018) 18(1):511. 10.1186/s12884-018-2160-z 30594169 PMC6311044

