# Supplementary material for: Endometrial scratching during hysteroscopy in women undergoing in vitro fertilization: a systematic review and meta-analysis
Source: Front Surg. 2023 Sep 19;10:1225111. doi: 10.3389/fsurg.2023.1225111 (PMC10546045; doi:10.3389/fsurg.2023.1225111)
Supplement: Supplementary file 1 [file Datasheet1.docx]

Supplementary Material

Endometrial injury during hysteroscopy in women undergoing in vitro fertilization: A systematic review and meta-analysis

**Evangelos Papanikolaou, Nikolaos Peitsidis*, Ioannis Tsakiridis, Georgios Michos, Antonios Skalias, Dimitrios Patoulias, Alexandros Poutoglidis, Apostolos Mamopoulos, Apostolos Athanasiadis, Grigorios Grimpizis, Robert Najdecki**

*** Correspondence:** Nikolaos Peitsidis: [nickpeits@windowslive.com](mailto:nickpeits@windowslive.com)


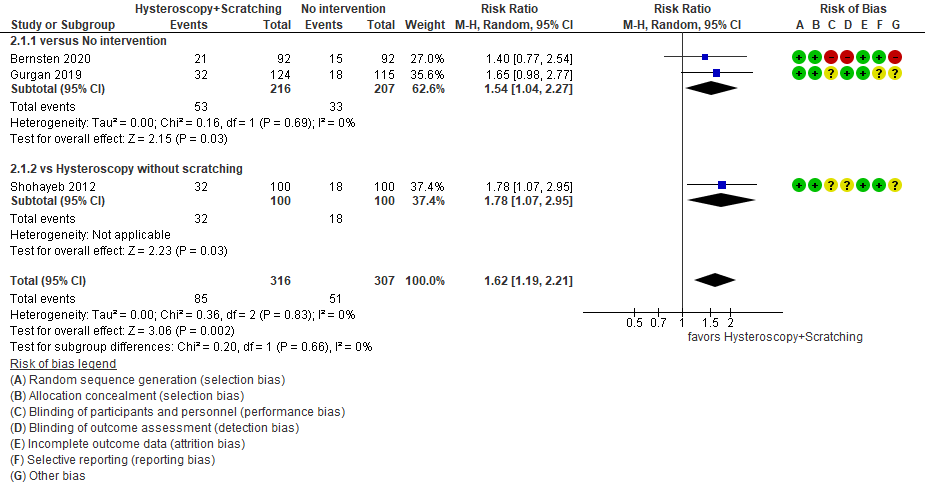
 **Supplementary Figure 1.** Sensitivity analysis of CPR including only RCTs


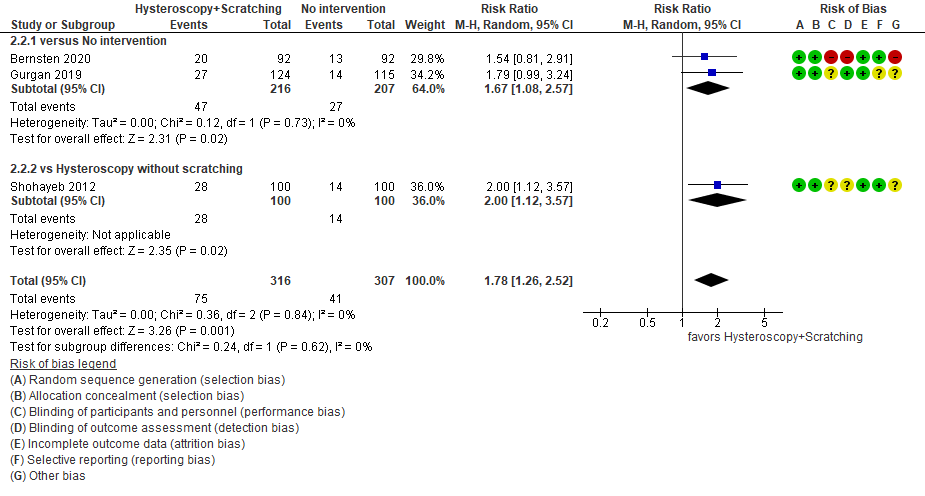
 **Supplementary Figure 2.** Sensitivity analysis of LBR including only RCTs


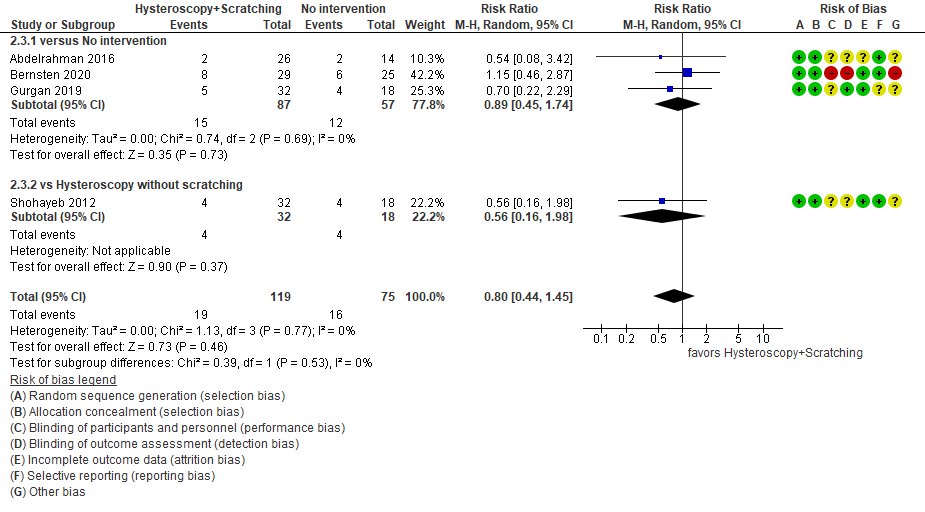
 **Supplementary Figure 3.** Sensitivity analysis of MR including only RCTs


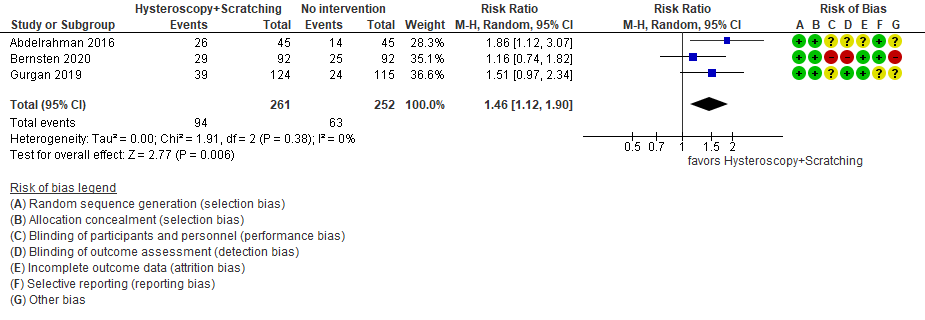
 **Supplementary Figure 4.** Sensitivity analysis of bHCG including only RCTs


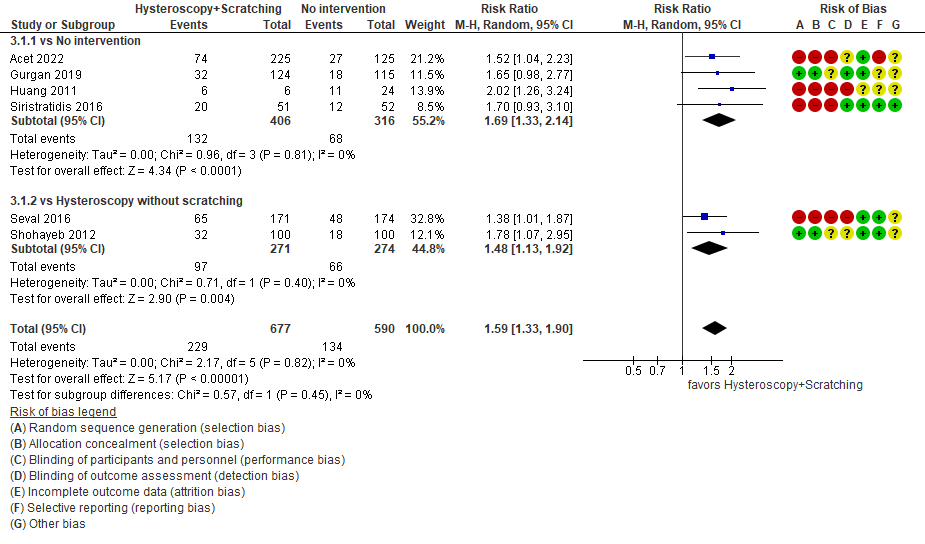
 **Supplementary Figure 5.** Subgroup analysis of CPR including only RIF patients


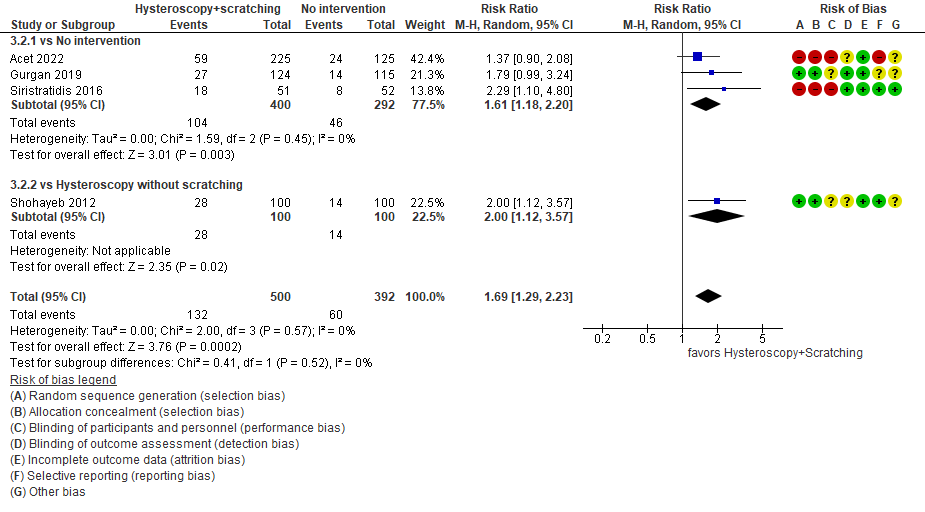
 **Supplementary Figure 6.** Subgroup analysis of LBR including only RIF patients


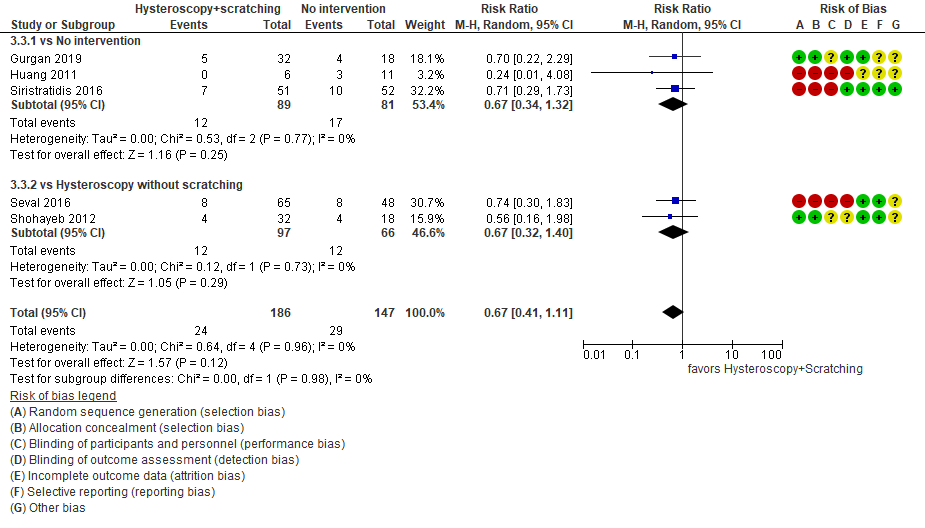
 **Supplementary Figure 7.** Subgroup analysis of MR including only RIF patients


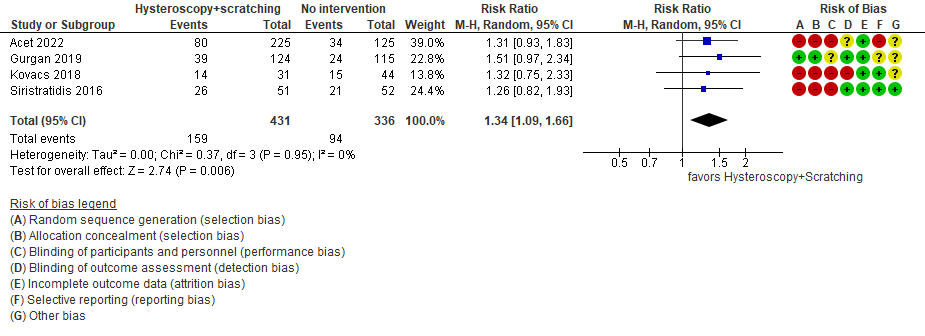
 **Supplementary Figure 8.** Subgroup analysis of bHCG including only RIF patients

**Supplementary Table 1.** Search strategy for each database

| Medline, PMC (through PubMed)  (endometrial injury OR endometrial scratching OR endometrial biopsy OR endometrial sampling OR endometrial damage) AND (infertility OR pregnancy OR live birth OR IVF OR ICSI OR ART OR assisted reproductive techniques OR embryo transfer OR embryo implantation OR endometrial receptivity) AND (hysteroscopy OR diagnostic hysteroscopy OR office hysteroscopy OR hysteroscopic evaluation OR hysteroscopy with or without endometrial scratching OR hysteroscopy with endometrial injury) |
| --- |
| ScienceDirect (only articles)  (endometrial injury OR endometrial scratching) AND (pregnancy OR live birth OR assisted reproductive techniques OR embryo transfer OR embryo implantation OR endometrial receptivity) AND hysteroscopy |
| Scopus  ("endometrial injury" OR "endometrial scratching" OR "endometrial biopsy" OR "endometrial sampling" OR "endometrial damage" ) AND (infertility OR pregnancy OR "live birth" OR IVF OR ICSI OR ART OR "assisted reproductive techniques" OR "embryo transfer" OR "embryo implantation" OR "endometrial receptivity" ) AND (hysteroscopy OR "diagnostic hysteroscopy" OR "office hysteroscopy" OR "hysteroscopic evaluation" OR "hysteroscopy with" OR "without endometrial scratching" OR "hysteroscopy with endometrial injury" ) |
| CENTRAL  ("endometrial injury" OR "endometrial scratching" OR "endometrial biopsy" OR "endometrial sampling" OR "endometrial damage" ) AND (infertility OR pregnancy OR "live birth" OR IVF OR ICSI OR ART OR "assisted reproductive techniques" OR "embryo transfer" OR "embryo implantation" OR "endometrial receptivity" ) AND (hysteroscopy OR "diagnostic hysteroscopy" OR "office hysteroscopy" OR "hysteroscopic evaluation" OR "hysteroscopy with" OR "without endometrial scratching" OR "hysteroscopy with endometrial injury" ) |
| Google Scholar (retrieval of first 900)  (endometrial injury OR endometrial scratching OR endometrial biopsy OR endometrial sampling OR endometrial damage) AND (infertility OR pregnancy OR live birth OR IVF OR ICSI OR ART OR assisted reproductive techniques OR embryo transfer OR embryo implantation OR endometrial receptivity) AND (hysteroscopy OR diagnostic hysteroscopy OR office hysteroscopy OR hysteroscopic evaluation OR hysteroscopy with or without endometrial scratching OR hysteroscopy with endometrial injury) |
